# Reconstituting the genome of a young allopolyploid crop, *Brassica napus,* with its related species

**DOI:** 10.1111/pbi.13041

**Published:** 2019-01-07

**Authors:** Dandan Hu, Wenshan Zhang, Yikai Zhang, Shihao Chang, Lunlin Chen, Yingying Chen, Yongdi Shi, Jinxiong Shen, Jinling Meng, Jun Zou

**Affiliations:** ^1^ National Key Laboratory of Crop Genetic Improvement College of Plant Science & Technology Huazhong Agricultural University Wuhan China

**Keywords:** *Brassica*, genetic diversity, genome evolution, introgression, recurrent selection

## Abstract

*Brassica napus* (A^n^A^n^C^n^C^n^) is an important worldwide oilseed crop, but it is a young allotetraploid with a short evolutionary history and limited genetic diversity. To significantly broaden its genetic diversity and create a novel heterotic population for sustainable rapeseed breeding, this study reconstituted the genome of *B. napus* by replacing it with the subgenomes from 122 accessions of *Brassica rapa* (A^r^A^r^) and 74 accessions of *Brassica carinata* (B^c^B^c^C^c^C^c^) and developing a novel gene pool of *B. napus* through five rounds of extensive recurrent selection. When compared with traditional *B. napus* using SSR markers and high‐throughput SNP/Indel markers through genotyping by sequencing, the newly developed gene pool and its homozygous progenies exhibited a large genetic distance, rich allelic diversity, new alleles and exotic allelic introgression across all 19 AC chromosomes. In addition to the abundant genomic variation detected in the AC genome, we also detected considerable introgression from the eight chromosomes of the B genome. Extensive trait variation and some genetic improvements were present from the early recurrent selection to later generations. This novel gene pool produced equally rich phenotypic variation and should be valuable for rapeseed genetic improvement. By reconstituting the genome of *B. napus* by introducing subgenomic variation within and between the related species using intense selection and recombination, the whole genome could be substantially reorganized. These results serve as an example of the manipulation of the genome of a young allopolyploid and provide insights into its rapid genome evolution affected by interspecific and intraspecific crosses.

## Introduction

The introgression of genomic regions from related species into a target crop has been used frequently for germplasm innovation, not only for a single locus but also at the whole‐genome level (Becker *et al*., 1995; Dhaliwal *et al*., 2017; Kuligowska *et al*., 2016; Mallet, 2005; Multani *et al*., 2003; Yu *et al*., 2012; Zamir, 2001; Zhan *et al*., 2017). The extensive introgression of exotic genomic regions into the gene pool can significantly enrich genetic diversity, lead to rapid structural genomic changes, increase the genetic distance and promote heterosis between the hybrid parents (Fu *et al*., 2012; Girke *et al*., 2012; Hurgobin *et al*., 2018; Jesske *et al*., 2013; Kovacs *et al*., 1998; Wang *et al*., 2005; Zou *et al*., 2011). Successful introgression from related species always relies on good interspecific crossing ability and efficient selection of the offspring to overcome ‘hybridization barriers’, ‘violent segregation’ and linkage drags (Jiang *et al*., 2007; Lewis *et al*., 2007). Therefore, creating a novel gene pool and improving the approach of the construction of this gene pool would provide insight into strategies for crop genetic improvement.

*Brassica napus* (A^n^A^n^C^n^C^n^) is an important oilseed crop grown worldwide but is a relatively young species derived from natural interspecific crosses of two diploid progenitors, *B. rapa* (A^r^A^r^) and *Brassica oleracea* (C^o^C^o^), approximately 7.5 thousand years ago; however, *B. napus* was only domesticated as an oilseed crop approximately 400 years ago and therefore, has a very short evolutionary and domestication history (Chalhoub *et al*., 2014). No wild varieties of *B. napus* have been found to date (Prakash *et al*., 2011). As a result, *B. napus* has very limited genetic diversity and high linkage disequilibrium within the genome, particularly in its C genome (Bus *et al*., 2011; Chen *et al*., 2008; Gyawali *et al*., 2013; Lombard *et al*., 2000; Qian *et al*., 2014; Wang *et al*., 2014b). The genus *Brassica*, consisting of six species, including *B. napus*, is comprised of A, B and C basic genomes, which can be divided into different subgenomes (A^r^/A^n^/A^j^, C^n^/C^o^/C^c^ and B^j^/B^c^/B^n^), and harbours rich variation not only among the genomes but also among the subgenomes (Chalhoub *et al*., 2014; Cheng *et al*., 2016; Liu *et al*., 2014; Navabi *et al*., 2013; Parkin *et al*., 2014; Pires *et al*., 2004; Warwick *et al*., 2006; Yang *et al*., 2016; Zou *et al*., 2016). To utilize the variation among the different basic genomes and different subgenomes to broaden the genetic diversity of *B. napus*, many studies have introgressed genomic regions from a single related species and even other genera, such as *B. rapa* (Qian *et al*., 2005), *B. oleracea* (Li *et al*., 2014; Quazi, 1988), *Brassica juncea* (Roy, 1984), *B. carinata* (Navabi *et al*., 2010, 2011), *Brassica maurorum* (Chrungu *et al*., 1999), *Sinapis arvensis* (Hu *et al*., 2002) and *Isatis indigotica* (Kang *et al*., 2017). In addition, substantial efforts have been extended to resynthesize *B. napus* by combining the genomes from two or more species, such as crossing *B. rapa* with *B. oleracea* (Becker *et al*., 1995; Hansen and Earle, 1994; Nagaharu, 1935), *B. juncea* with *B. carinata* (Chatterjee *et al*., 2016), and *B. carinata* with *B. rapa* (Li *et al*., 2004, 2006; Xiao *et al*., 2010).

A new type of *B. napus* was developed with 20%–40% of the subgenomic introgression from *B. rapa* or the combination of *B. rapa* and *B. carinata*. These lines were called the first generation of the new‐type of *B. napus* (G1), and the intersubgenomic hybrids developed with the natural *B. napus* demonstrated strong heterosis in seed yield (Li *et al*., 2004, 2006; Qian *et al*., 2005, 2006). Hundreds of lines with improved genetic characteristics and exotic subgenomic components were bred as the second generation of the new‐type of *B. napus* (G2) from intercrosses between selected G1 lines; these G2 lines exhibited stronger heterosis when crossed with natural *B. napus*, and the seed yield heterosis of the hybrids was positively correlated with the content of the A^r^C^c^ subgenome in the new‐type *B. napus* (Li *et al*., 2006; Zou *et al*., 2010). The G1 and G2 lines were distantly separated from the traditional cultivars of *B. napus* with significantly higher richness of the specific alleles than that in the accessions of *B. napus* collected internationally, and they formed a unique subpopulation (Chen *et al*., 2010). However, these G1 and G2 lines were developed based on a few parental *B. rapa* and *B. carinata* accessions, and the subgenomic variation within these two species (*B. rapa* and *B. carinata*) could be further introgressed.

To further introgress the A^r^C^c^ subgenomic variation within the parental species into *B. napus*, a population of new‐type *B. napus* (third generation, G3) was constructed by successively crossing selected new‐type *B. napus* (G2) lines (75% of A^r^/C^c^ exotic introgression on average) with 436 artificial *Brassica* hexaploid plants (A^r^A^r^B^c^B^c^C^c^C^c^) that involving the genomic components of 74 accessions of *B. carinata* and 122 cultivars of *B. rapa*. The construction of *B. napus* was followed by extensive selection assisted with simple sequence repeat (SSR) and amplified fragment length polymorphism (AFLP) markers (Jiang *et al*., 2007; Xiao *et al*., 2010; Zou *et al*., 2010, 2018). The G3 population consisting of two subpopulations, the Poly‐C^c^ subpopulation (polymorphic in the C subgenome) and the Poly‐A^r^ subpopulation (polymorphic in the A subgenome), demonstrated not only relative genetic stability with a normal chromosome constitution (2n = 38, AACC) checking by cytogenetic observation on somatic cells and pollen mother cells with chromosome counting and GISH (genomic in situ hybridization), morphological observation on plant fertility and seed setting set, and genotyping with an Illumina *Brassica* 60K AC SNP chip array but also rich genetic diversity and abundant genomic variation, such as the exotic introgressions from *B. carinata* and *B. rapa* (87.2% on average), allelic combinations, and reconstructed linkage disequilibrium patterns and haplotype blocks (Xiao *et al*., 2010; Zou *et al*., 2018). However, intensive selection with self‐pollination for each single G3 inbred line might limit the recombination and appearance of novel genetic variation and regions with strong linkage disequilibrium and selective sweeps (Zou *et al*., 2018).

Novel genetic variation could be incessantly generated by genome shuffling and new recombination. To maintain and create additional novel genetic diversity, this study demonstrated a strategy to reorganize the genome of *B. napus* by creating a dynamic, novel breeding gene pool of the G3 new‐type *B. napus* with improved traits; these lines were achieved through extensive recombination within and between a Poly‐A^r^ subpopulation and Poly‐C^c^ subpopulation with the assistance of a mating system of dominant genic male sterility (DGMS) (Li *et al*., 1985; Liu *et al*., 2008) by facilitated intercrossing among different RS (recurrent selection) lines in each cycle and five rounds of recurrent selection on a range of important traits. With this gene pool, we evaluated (i) the genome‐wide genetic diversity and exotic introgression; (ii) the efficiency of intensive selection and recombination to improve agronomic traits and seed quality traits, and (iii) the variation in favourable traits. This study provides a reference to construct novel gene pools of crops and brings crop germplasm innovation closer to routine breeding practices. The results provide novel insights into how to rapidly reorganize the genome of a young allopolyploid crop based on genome plasticity and into the consequences of intensive selection and recombination on rapid genomic reorganization.

## Results

### Creating a dynamic breeding gene pool of the new‐type *B. napus*

**Figure 1:**
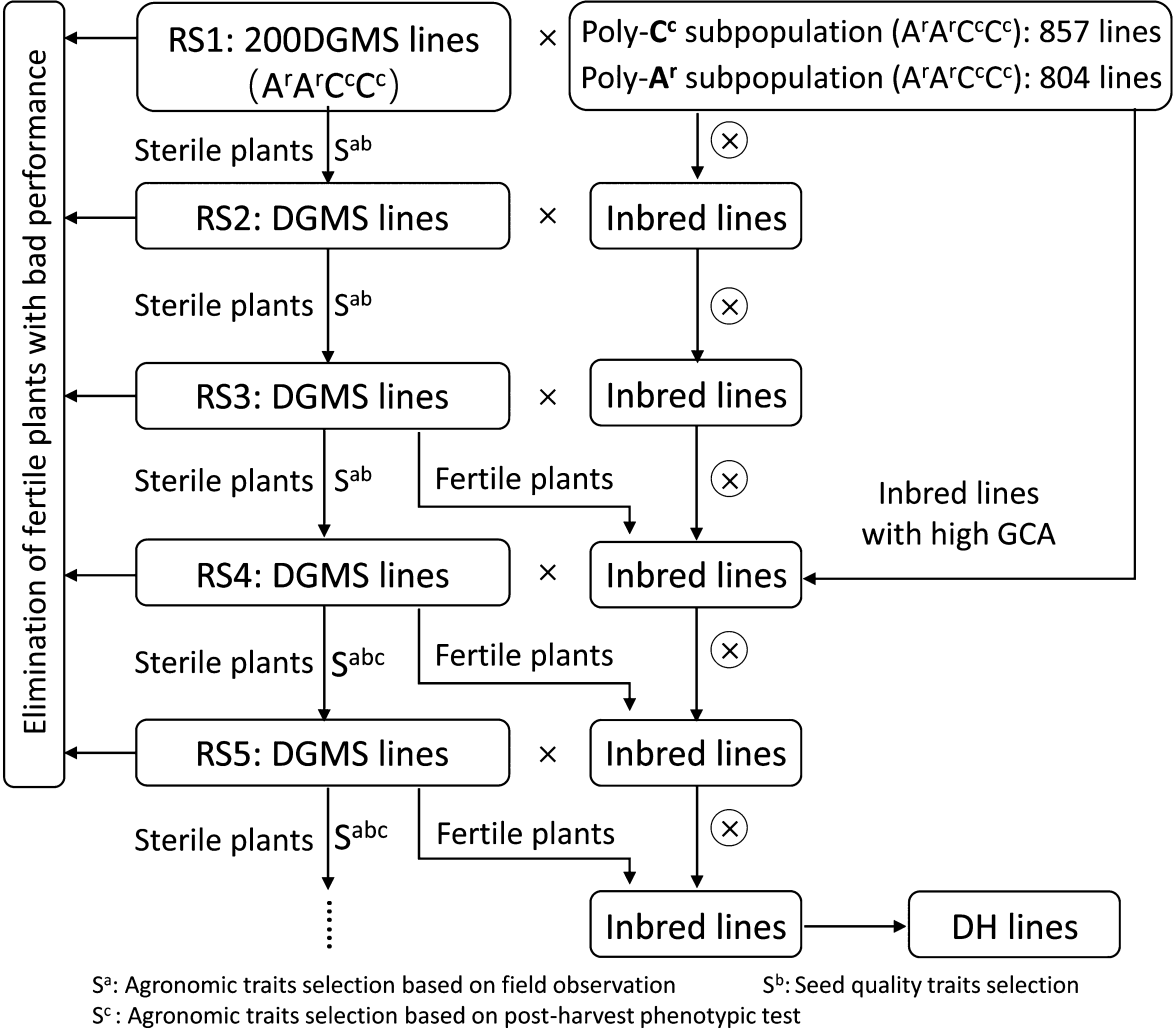
The process for developing a gene pool of new‐type *Brassica napus* (A^r^A^r^C^c^C^c^) with five rounds of recurrent selection. The new‐type of DGMS lines were interplanted with inbred lines from the Poly‐A^r^ subpopulation and the Poly‐C^c^ subpopulation in a net shed with the latter serving as the supplier of pollen and bees serving as the pollinators. The Poly‐C^c^ subpopulation involves introgression from 72 *B. carinata* accessions and 25 *B. rapa* accessions (Xiao *et al*., 2010). The Poly‐A^r^ subpopulation involves introgression from seven *B. carinata* accessions and 111 *B. rapa* accessions (Zou *et al*., 2018).

To develop a dynamic gene pool of the new‐type *B. napus* (A^r^A^r^C^c^C^c^) with rich genetic diversity and favourable traits related to seed yield and edible quality, we used a dominant genic male system (DGMS) with the new‐type *B. napus* genetic background to perform recurrent selection (RS) to incorporate the genetic diversity harboured in two subpopulations of new‐type *B. napus* from crosses of 7 *B. carinata* with 111 *B. rapa* and 25 *B. rapa* with 72 *B. carinata*, specifically, a Poly‐A^r^ subpopulation and a Poly‐C^c^ subpopulation, respectively (Figure 1, Figure S3). See the Methods for more detail about the development of the DGMS new‐type *B. napus* lines and two subpopulations.

A total of 804 F_3_ lines and 857 F_4_ lines representing the Poly‐A^r^ and Poly‐C^c^ subpopulations, respectively, were interplanted with 200 new‐type DGMS lines as the founder parents (Figure 1). After five rounds of RS, most plants of the RS population were expected had combined subgenomes of the two polymorphic subpopulations and achieved extensive outcrossing of the two polymorphic subpopulations. Thus, frequent recombination was expected to have occurred, and abundant genetic variation would be introduced during this process. Ultimately, we constructed a highly heterozygous breeding gene pool of new‐type *B. napus* with exogenous introgression from 74 accessions of *B. carinata* and 122 accessions of *B. rapa*.

### Population genetic diversity and novel genomic variation in the recurrent selection population and its derived double haploid lines

**Figure 2:**
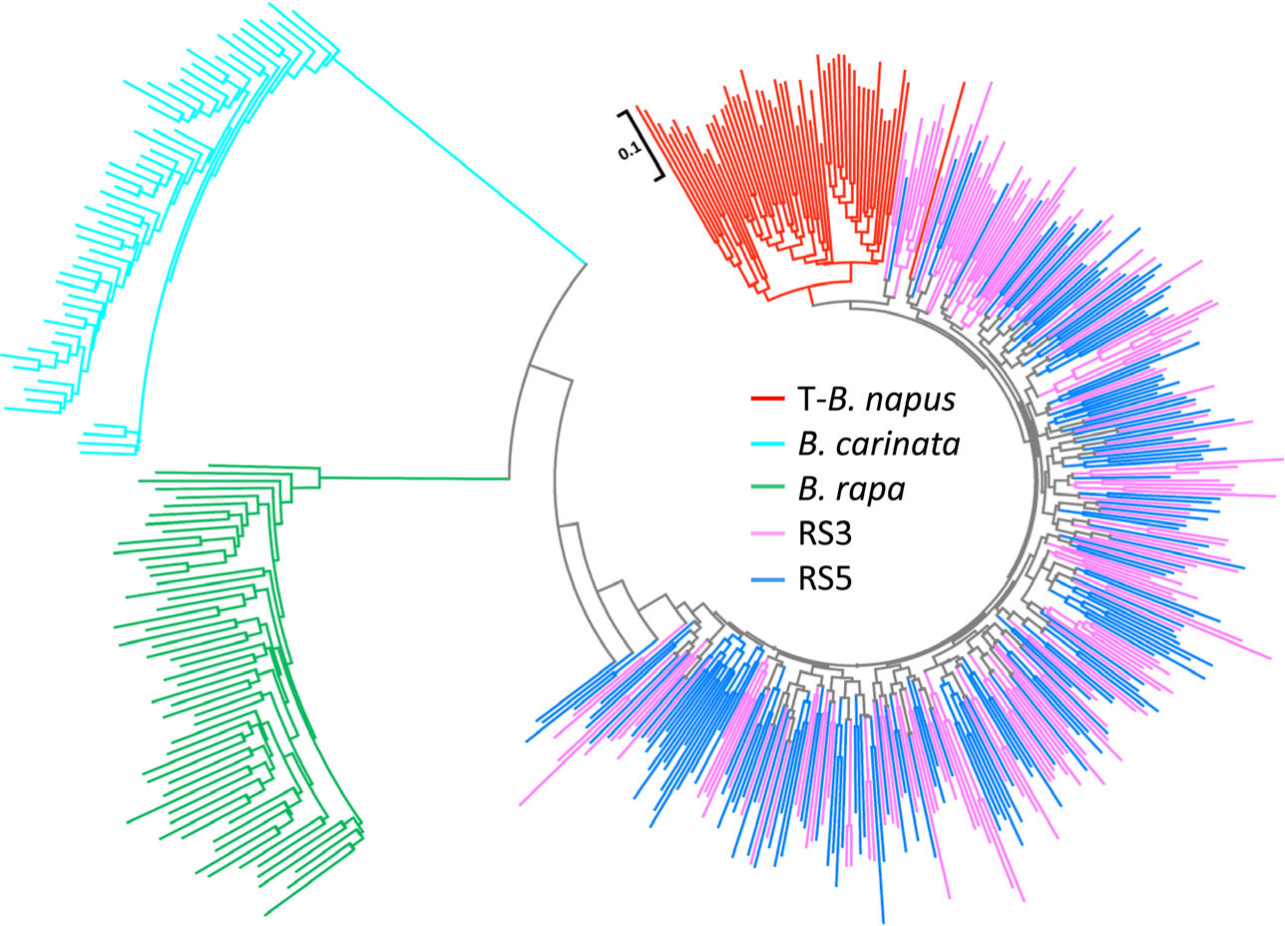
Genetic clustering of 486 *Brassica* accessions. Neighbour‐Joining tree of the investigated 486 *Brassica* accessions from RS3 (third round of recurrent selection population, 160 accessions), RS5 (fifth round of the recurrent selection population, 160 accessions), T‐ *Brassica napus* (traditional *B. napus*, 56 accessions), *B. carinata* (55 accessions) and *B. rapa* (55 accessions) subpopulations based on Nei's genetic distance with 152 loci.

A total of 486 *Brassica* accessions (Table S1), 160 from RS3 (randomly selected from 80 lines), 160 from RS5 (randomly selected from 80 lines), 56 from T‐*B. napus* (traditional *B. napus*), 55 from *B. carinata* and 55 from *B. rapa* were evaluated with 82 markers, including 80 SSR markers and 2 Indel markers, that covered the A and C genomes to examine multiple alleles and heterozygote allelic combinations (Table S2). One hundred and fifty‐two loci with 615 alleles were detected. Although most of the amplified alleles could be attributed to the specific loci of the A (73) and C genomes (59), alleles were also amplified from three specific loci of the B genome, and 17 other loci were also attributed to the A and B genomes. A very slight decrease in allelic diversity occurred from the relatively early generation, RS3 (0.41), to the later generation, RS5 (0.40), but both subpopulations were divided into one genetic cluster and demonstrated very similar genetic diversity and differences with the parental species (Table 1). In addition, their total number of alleles and the genetic diversity were higher than those of the T‐*B. napus*, particularly in the C genome. The heterozygosity of the RS3 and RS5 subpopulations were more than twofold higher than that of T‐*B. napus* (Table 1, Figure S1), and the high heterozygosity indicated full intercrossing among the RS lines indicating high recombination within the RS population. As shown in the Neighbour‐Joining tree (Figure 2), the RS3/RS5 subpopulations and traditional *B. napus* separated as one large genetic cluster from the other two species, *B. carinata* and *B. rapa*, due to species differentiation. Although they belong to one species, the RS3/RS5 subpopulations and the traditional *B. napus* also separated from one another into two different genetic clusters.

**Table 1:** Genetic diversity of each subpopulation

Markers	Materials	Allele/locus	Gene Diversity	Heterozygosity	Genetic distance	
					Within population	T‐*B. napus*
Whole‐genome loci	*B. carinata*	1.81	0.15	0.01	0.07–0.29	‐
	*B. rapa*	2.77	0.28	0.05	0.11–0.46	‐
	T‐*B. napus*	2.99	0.38	0.04	0.24–0.84	‐
	RS3	3.16	0.41	0.10	0.11–0.81	0.32–0.89
	RS5	3.15	0.40	0.11	0.21–0.74	0.32–0.84
A genome loci	*B. rapa*	3.76	0.44	0.08	0.19–0.81	‐
	T‐*B. napus*	3.04	0.40	0.03	0.22–0.94	‐
	RS3	3.12	0.40	0.09	0.13–0.95	0.25–0.97
	RS5	3.07	0.39	0.10	0.16–0.84	0.23–0.96
C genome loci	*B. carinata*	2.59	0.30	0.02	0.13–0.65	‐
	T‐*B. napus*	2.90	0.35	0.05	0.14–0.72	‐
	RS3	3.24	0.42	0.11	0.08–0.93	0.18–0.99
	RS5	3.27	0.41	0.12	0.15–0.90	0.20–0.93

*B. carinata* represents 55 *B. carinata* accessions; *B. rapa* represents 55 *B. rapa* accessions; T‐*B. napus* represents 56 traditional *B. napus* accessions; RS3 represents 160 accessions of the third round of recurrent selection population; RS5 represents 160 accessions of the fifth round of the recurrent selection population. ‘Allele/locus’, ‘Gene Diversity’ and ‘Heterozygosity’ of the RS3 and RS5 subpopulations were calculated based on 56 accessions randomly selected from each subpopulation respectively.

**Figure 3:**
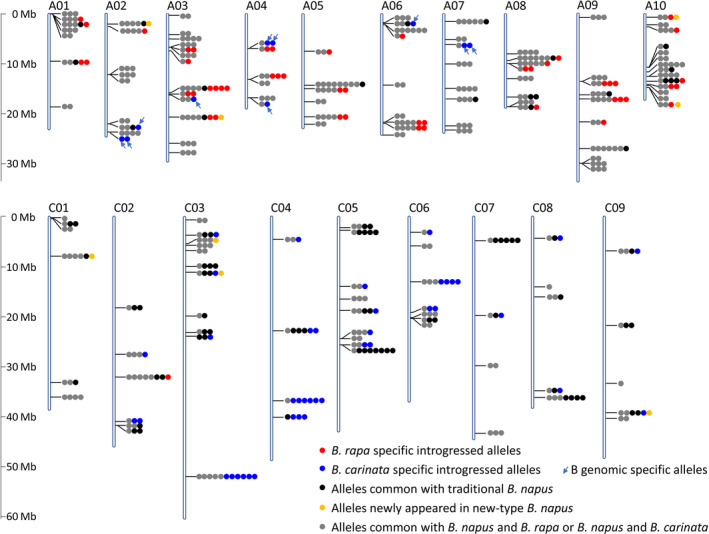
Distribution of the 543 alleles detected in the recurrent selection population. Markers represents 147 loci were distributed across all the chromosomes of *Brassica napus*. The Darmor‐*bzh* genome (v4.1) was used as the reference genome to align the sequences of the markers. A total of 30, 29 and 8 loci were detected with alleles specially from *B. rapa, B. carinata*, and newly appeared alleles, respectively.

A total of 147 loci with 543 alleles were detected in the new‐type *B. napus*; in the 320 RS plants, 40.1% of the loci (59) were detected with one to six alleles that could be identified as specific introgression from the parental species, *B. carinata* (B^c^B^c^C^c^C^c^, 29 loci) and *B. rapa* (A^r^A^r^, 30 loci) (Figure 3). Twenty‐five of the 59 loci contained more than one *B. carinata*/*B. rapa*‐specific allele, indicating that the genetic variants within the parental species were introgressed to the gene pool through interspecific crossing. Exotic allelic introgression from *B. carinata* included not only allelic introgression from the C^c^ genome (22 loci with 35 alleles) but also 10 alleles (detected in seven loci) from the B^c^ genome of *B. carinata*, which were detected along with the alleles amplified at seven homologous loci in the A genome, and the B^c^ genome introgressed alleles were exhibited with their homologous alleles in the A genome (A02, A03, A04, A06 and A07) (Figure 3). In addition to direct exotic introgression, novel alleles that specifically appeared in new‐type *B. napus* were detected in eight loci.

**Figure 4:**
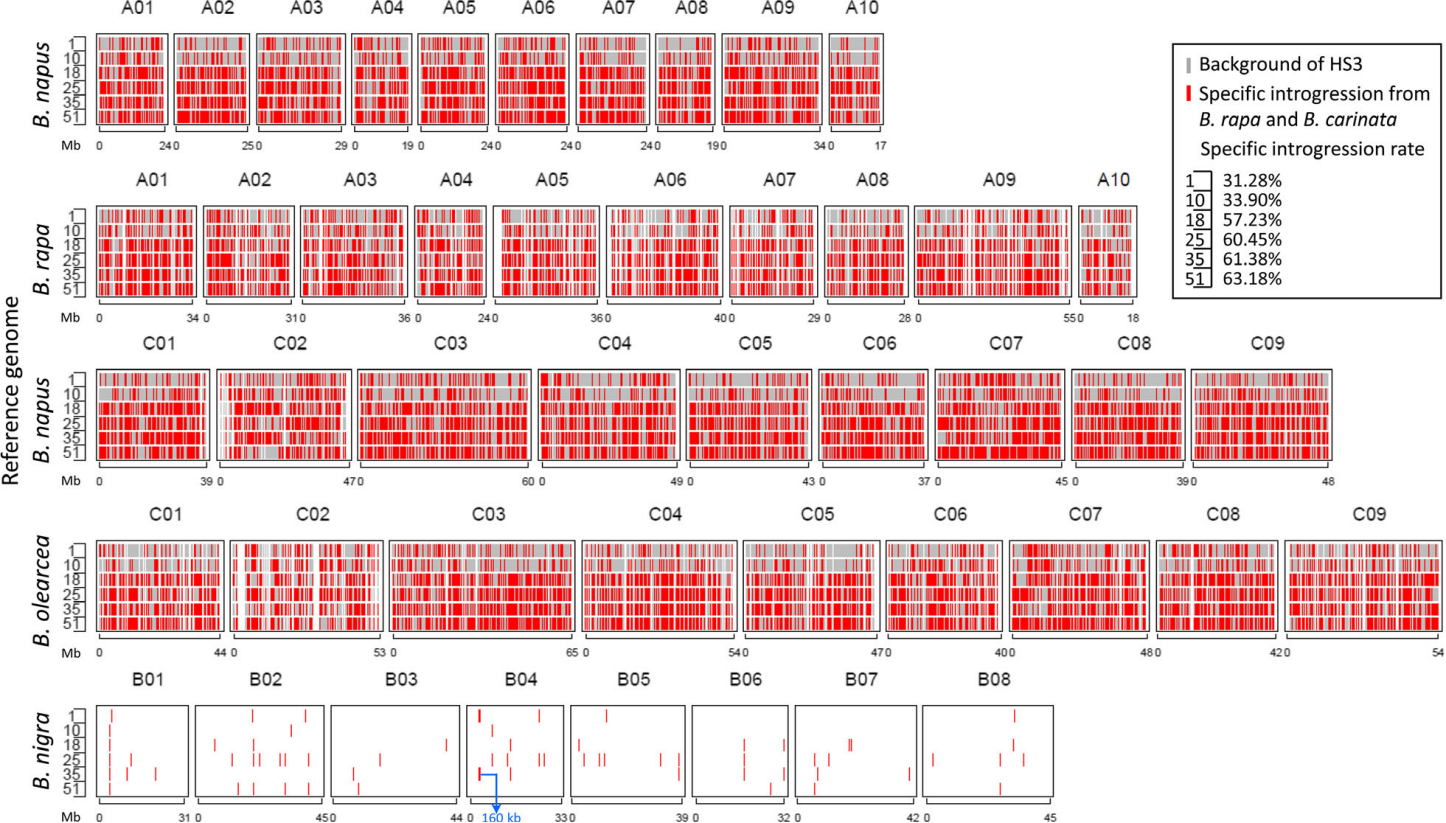
Demonstration of specific genomic introgression in six new‐type *Brassica napus* DH lines. Gray lines indicate that the allele detected in the new‐type *B. napus* DH lines is the same as that in HS3, and red lines indicate that the allele detected in the new‐type *B. napus* DH lines is different from that in HS3. A blue arrow indicates a 160 kb B genome region identified in the new‐type *B. napus* DH lines.

Hundreds of double haploid (DH) lines (893) were developed from the selected fertile lines of the RS population using microspore culture (Figure 1), and 51 DH lines were selected to evaluate the genome‐wide variation fixed in these homozygote lines compared with 10 representative traditional *B. napus* lines and the founder *B. napus* parent of the new‐type *B. napus*, Huashuang 3 (HS3), by genotyping by sequencing (GBS) (Table S1). Based on the reference genome of *B. napus* (Darmor‐*bzh*, V4.1), we detected 15,937 A and 20,415 C genomic SNP/Indel markers with missing rates less than 50%. An additional set of markers with 4,977 A, 8,503 C and 390 B genomic SNP/Indel markers was detected when using *B. rapa*,* B. oleracea* and *B. nigra* as the reference genome respectively. Finally, a total of 50,222 high‐quality markers (44,124 SNP markers and 6,098 Indel markers) were used to determine the specific introgression in the new‐type *B. napus* DH lines. On average, 51.99% of the genome (varying from 31.28 to 63.18%) of different lines within the population were different from those of their parent, HS3. Combining the genomic variation across the whole population, approximately 99.63% of the genome harboured variation different from HS3 as the result of introgression from *B. carinata* and *B. rapa* (Figure 4, Figure S2). Small but obvious signals were detected in the 140 B genome introgressions represented by 390 B genome‐specific markers in the genome of the new‐type *B. napus* DH lines, and most of the B genome introgressions were small segments traced by only one or several markers, except for one comparatively large segment covering 160 kb in chromosome B04 (3,563,715 ‐ 3,723,750) and containing 12 annotation genes involved in the biological process of mitotic G1 and G2 phase, microtubule bundle formation, embryo and flowering development (Figure 4, Figure S2; Tables S3, S4).

### Trait improvement of the gene pool by recurrent selection

Considering the relatively poor traits of interspecific offspring in the early generations, we performed a relatively permissive selection of morphology and focused on seed setting when harvesting in the first three rounds of RS (RS1, RS2 and RS3) and continuously increased the population size to allow more recombination before later selection on yield‐related traits. Field selection on the agronomic traits and indoor selection on the seed quality traits were performed in all the five rounds of selection, and the indoor selection on the agronomic traits based phenotypic test was started from the fourth rounds of selection (Figure 1). We collected data on the seed quality traits of each harvested sterile line in the five rounds of RS and the data of the agronomic traits of each harvested sterile line in the last two rounds of RS (RS4 and RS5). The seed glucosinolate content (GSLC) and erucic acid content (EAC) decreased dramatically from 75.8% to 31.1% and from 9.4% to 0.8% respectively. Overall, the total oil content (OC) increased by 3.32% but decreased in RS3 and RS4. For the agronomic traits, the seed number per pod (SN) and thousand seed weight (TSW) increased 33.2% and 13.9% from the RS4 to RS5 subpopulations respectively (Table S5). Although these trait data were collected in different years and might be affected by environmental factors, trait improvement, specifically in seed quality traits and rich phenotypic variation were exhibited across the population through the assessment of a total of 3007 lines (Table S5).

**Figure 5:**
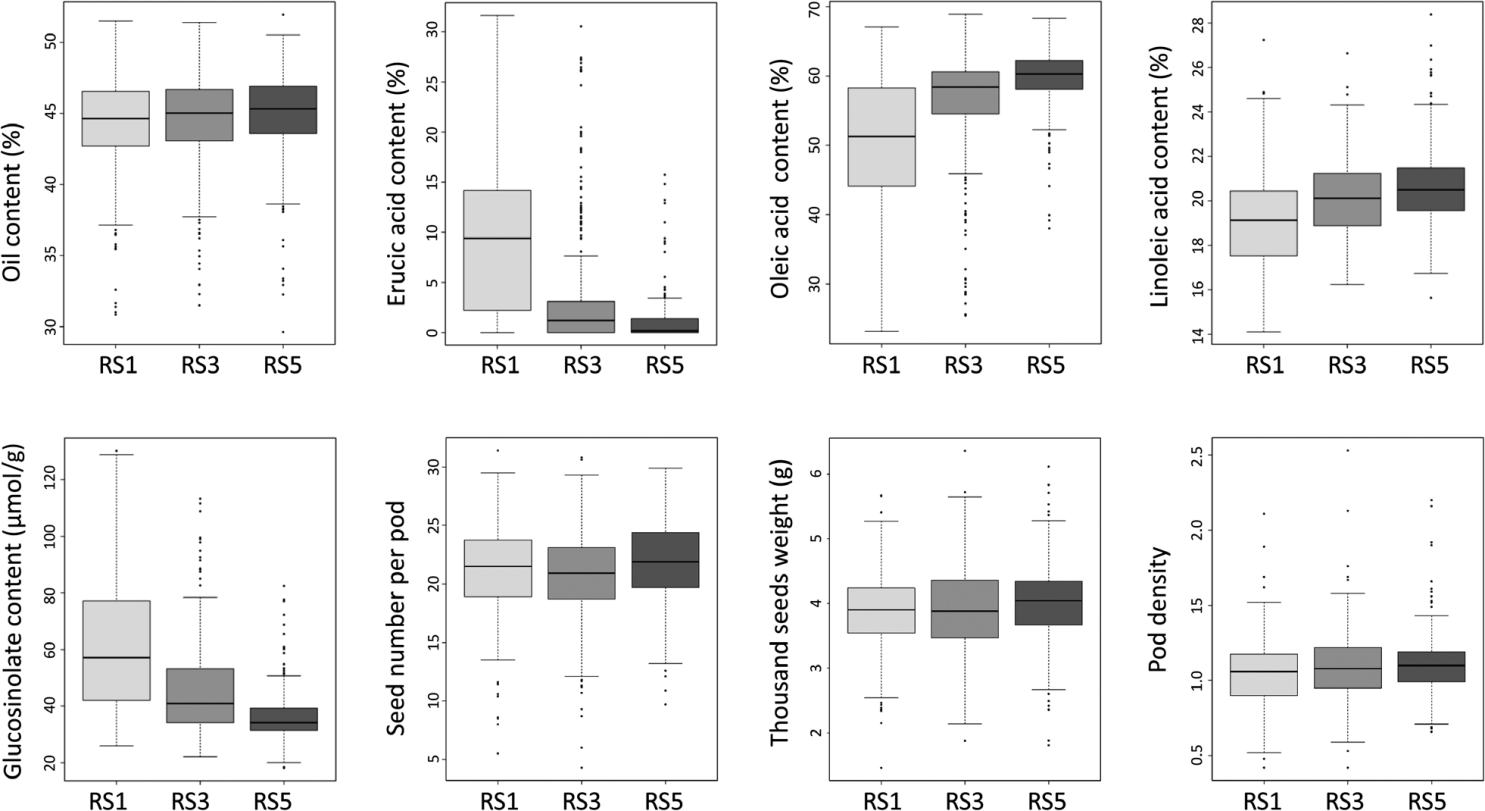
Boxplot for the seed quality and agronomic traits of different generations of the recurrent selection population.

To further evaluate the improvement in traits in the RS subpopulation, a total of 240 lines (80 lines of each generation) were randomly selected from the RS1, RS3 and RS5 subpopulations and were planted in the same environment. For seed quality, the difference in the OC among the three generations was not significant, but the difference in EAC, oleic acid content (OLE), linoleic acid content (LEI) and GSLC among the three generations was significant (*P* < 0.01). The average seed oil content increased slowly (from 44.2% to 45.0%) from RS1 to RS5, but the variation obviously increased with the maximum and minimum oil content of seeds reaching 52.0% and 32.0%, respectively (Figure 5). The EAC was reduced from 9.4% to 1.0%, resulting in an increase in the OLE from 50.2% to 59.8%, and the LEI increased from 19.1% to 20.6% with the highest contents of OLE and LEI reaching 68.9% and 28.4% respectively. The average seed GSLC was significantly reduced from 60.9 μmol/g in the RS1 subpopulation to 36.2 μmol/g in the RS5 subpopulation, and most individuals of the RS5 subpopulation were of canola quality with low GSLC and EAC in the seeds of the *Brassica* oilseed crop (Figure 5).

In terms of the agronomic traits, the difference in the TSW (*P* < 0.05), pod density (PD) (*P* < 0.01) and SN (*P* < 0.01) among the three generations was significant. The TSW and PD continually increased from RS1 to RS5, but the SN only increased from RS3 to RS5 (Figure 5). The improvement in the TSW, PD and SN from the third generation to the fifth generation reached 2.6, 5.7% and 3.3%, respectively, and the maximum values of TSW and PD were 6.1 g and 2.2 respectively (Table S6). However, a decrease in the maximum value of the SN from 31.4 to 29.9 was also observed. In general, the three agronomic traits were particularly improved under stronger and more accurate selection in RS4 and RS5. For example the yield‐related trait SN increased 5.3% from RS3 to RS5. These results further confirmed rapid trait improvement by the RS for the offspring derived from hundreds of interspecific crosses and rich, favourable phenotypic variation harboured in the gene pool, which could be controlled by the rich genomic variation and high frequency of favourable allelic variation in the gene pool.

## Discussion

Reconstituting the genome of a young allopolyploid species with its related species to create a novel crop breeding population not only significantly broadened the genetic diversity of this species but also accelerated genome evolution and reorganization. This approach provided us with many types of novel breeding germplasm for rapid trait improvement and a potential to develop a new heterotic population for hybrid breeding. Focusing on developing a novel, dynamic breeding population rather than on a few resynthesized lines, we could broaden the genetic variation as much as possible to optimize favourable and unique traits in the applied breeding practices. To accomplish this, we constructed a novel gene pool of *B. napus* (A^r^A^r^C^c^C^c^) based on one accession of *B. napus*, 74 accessions of *B. carinata,* and 122 accessions of *B. rapa* by incorporating thousands of lines of the Poly‐A^r^ and the Poly‐C^c^ subpopulations of the new‐type *B. napus* (Xiao *et al*., 2010; Zou *et al*., 2018) using the DGMS system followed by intensive recurrent selection (Figure 1). With this approach, novel genetic variation was generated from (i) hundreds of interspecific crossings when the Poly‐A^r^ and the Poly‐C^c^ subpopulations were created and interspecific‐crossing induced genomic changes, such as deletions, duplications, translocations and new allelic combinations; (ii) introgression between and within the related oilseed crops, that is 74 *B. carinata* and 122 *B. rapa* accessions and (iii) multiple rounds of recombination via recurrent selection assisted with the DGMS system.

Considering the detection of multiple alleles of the homologous loci and the high heterozygosity of the RS lines, A and C genomic SSR markers were selected based on the genetic map of *B. napus* and *B. carinata* (Guo *et al*., 2012; Luo *et al*., 2017) and traditional *B. napus*,* B. rapa* and *B. carinata* accessions were used as a reference to evaluate the heterozygote RS population. We observed rich genetic variation in the gene pool and significant divergence from the traditional *B. napus* (Figure 2, 3; Table 1). First, the gene pool exhibited great genetic diversity and large genetic distance, and this gene pool clustered into a group distinct from the traditional *B. napus* accessions from Asia, Europe, North America and Australia (Table 1; Table S1; Figure 2). The high heterozygosity of the RS5 subpopulation indicated successful hybridization during the recurrent selection and suggested rich recombination between the different subgenomes (Figure 3, Figure S1; Table 1). The gene diversity of the gene pool was higher than that of traditional *B. napus* accessions, particularly in the C genome. This is favourable for rapeseed breeding, because the genetic diversity in the C genome is very low in the traditional *B. napus* (Bancroft *et al*., 2011; Bus *et al*., 2011; Delourme *et al*., 2013; Rahman *et al*., 2011; Wang *et al*., 2014b; Zhao *et al*., 2013).

The gene pool contained unique genetic variation at the whole‐genome level. By comparison with the parents and accessions of the three parental species as controls, we distinguished the specific alleles amplified in the different subgenomes. For the heterozygote RS population, at least 17.5% of the 543 alleles detected in 147 loci in the gene pool were confirmed as specifically introgressed from *B. carinata* and *B. rapa*, and they were distributed in almost all the chromosomes. Many allelic variations within hundreds of the *B. carinata*/*B. rapa* accessions were also detected in the gene pool. However, the small number of the SSR markers and the lack of the founder parent of HS3 as a control might have led to the underestimation of the genomic variation. In addition, the genome‐wide estimation on 51 DH lines by 50,244 SNP/Indel markers generated from GBS was conducted and compared with HS3 (Figure 4, Figure S2). The published reference genome sequence of *B. carinata* was not currently available. Therefore, to detect the exotic introgression of *B. rapa* and *B. carinata*, we used the published genome sequences of *B. napus* (Chalhoub *et al*., 2014), *B. rapa* (Wang *et al*., 2011), *B. nigra* (Yang *et al*., 2016), and *B. oleracea* (Parkin *et al*., 2014) together as a reference to estimate the variation that originated from *B. rapa* and *B. carinata*. By comparison with HS3, we observed that approximately half of the genomic components of the new‐type *B. napus* DH lines were specifically introgressed from the founder parents *B. rapa* and *B. carinata*. Alternatively, an obvious but small amount of B genomic introgression was detected in the new‐type *B. napus* DH lines (Figure 4; Figure S2), and a 160 kb B genomic introgression region was detected with 12 annotation genes involved in the biological process of mitosis, embryo and flower development (Table S4), which may have some impacts for the genome stability and plant development of the new‐type *B. napus*. The SSR markers and GBS markers assisted with the genotype calling method with multiple species genomes as a reference enabled us to obtain more specific genomic markers, and in particular, we detected small segments of the B genome introgression (Figure 3, 4; Figure S2), which we could not distinguish when using the *Brassica* Infinium 60K SNP array and the *B. napus* as the only reference genome (Zou *et al*., 2018). Those lines with specific exogenous introgression would be ideal for future analyses of structural variation, genome stability and how those variations influence traits through genotyping by the newly developed *Brassica* Infinium 90K SNP array, which includes the SNP from the A/C/B genomes or the whole‐genome wide sequencing technology. For a more comprehensive analysis of the structural variation, we would also choose an appropriate marker distribution and density according to the linkage disequilibrium pattern across the different chromosomes. Generally, introducing genetic variation from the B genome of *B. carinata* to *B. napus* is very difficult. To date, only a few cases have successfully introgressed the B genome segments into *B. napus*, and it would be favourable to introduce the B genome segment to introduce resistance‐related genes to *B. napus* (Dhaliwal *et al*., 2017; Navabi *et al*., 2010, 2011; Xiao *et al*., 2010). Based on the results described above, the inference is that homologous exchange events occurred between the B and A/C genomes. In addition, brand‐new variation could be generated during the change from hexaploid (AABBCC) to tetraploid (AACC) because of chromosomal breakage or fusion, autosyndetic chromosome rearrangement, homologous exchange and retrotransposon activation as reported in previous studies of resynthesized *B. napus* (Hurgobin *et al*., 2018; Xiong *et al*., 2011; Zou *et al*., 2011, 2018). Rich new alleles and frequent deletion and duplication events were demonstrated in the inbred lines from the A^r^ and C^c^ polymorphism subpopulations (Xiao *et al*., 2010; Zou *et al*., 2011, 2018). We also detected eight alleles specifically in the RS population possibly because of induced novel alleles (Figure 3). In summary, this approach to synthesize new‐type *B. napus* through massive interspecific crossing between hundreds of *B. rapa* and *B. carinata* lines and intensive recombination and selection by recurrent selection was effective at broadening the genetic diversity and restructuring the whole genome of *B. napus*.

Rich phenotypic variation was also documented in the RS population. Almost all the traits examined showed extensive variation (Figure 5; Tables S5, S6). Many individuals with desirable traits, such as high oil content (up to 52%) and high unsaturated fatty acid (oleic acid) content (up to 68.94%), large seed (thousand seed weight up to 6.36 g) and high seed number per pod (31.4 seeds), were discovered (Figure 5; Table S6). Previous studies reported that inbred/DH lines with high nitrogen efficiency were selected from the gene pool (Wang *et al*., 2014a, 2015). These favourable variations may come from the founder parents, but they may also be caused by genomic changes, such as the chromosome rearrangement, homologous recombination, homoeologous shuffling, transposon activation and epigenetic modification. Desirable traits, such as the resistance to disease and pod shatter, that are harboured by the founder parents *B. carinata* and *B. rapa*, with high or low heritability, were introgressed to rapeseed easily or arduously through interspecific crossing (Dhaliwal *et al*., 2017; Diederichsen *et al*., 2009; Leflon *et al*., 2007; Raman *et al*., 2017). For example pod shatter resistance, which is a low heritability trait, was introduced to the *B. napus* along with the introgression of the B‐genome segments from *B. carinata*, and these introgression lines showed higher pod strength than *B. napus* parents; some lines were almost equal to that of the *B. carinata* parents (Dhaliwal *et al*., 2017). It is of high potential that these traits have been introgressed to the gene pool and could be exploited.

Interspecific crossing not only introgresses genetic variation underlying favourable traits but also brings unfavourable traits because of linkage drag from exotic species or from unfavourable genetic changes caused by recombination and structure variations (Jiang *et al*., 2007; Lewis *et al*., 2007). We aimed to introduce successive new recombination to form a dynamic diversity gene pool with rich genomic variation and favourable phenotypic variation as much as possible using recurrent selection. Therefore, the selection scheme and the male sterility system of the recurrent selection were of crucial importance to promote the recombination and genetic improvement of the gene pool. Considering the large segregation and relatively poor traits in the early generations, we adopted the simplest mass selection scheme (Sorrells and Fritz, 1982), which was easy and less time‐consuming for population improvement with only one selection per round in one year and no requirement for test crossing and the phenotypic identification of the progeny. We performed morphological selection in the field during the first three cycles and scored the seed quality trait data after we harvested the selected plants from the field. The selection on the first three cycles was relatively permissive to avoid strong selection pressure and bottleneck and promote full recombination within diverse genotypes to a broad genetic basis for later selections. As expected, five rounds of mass selection successfully yielded a highly heterozygous gene pool. All the target traits improved, and the difference among the generations of all the traits reached a significant level with exception of the OC (Figure 5). Comparatively, the improvements in the EAC and GSLC were the most rapid, because they have relatively high heritability with simple genetic architecture controlled by a few major genes. As a result, the rapid reduction in the EAC may promote the increase in oleic acid (Table S6), because erucic acid is synthesized with oleic acid as a substrate under the control of the *FAE1* gene (Han *et al*., 2001; Lassner *et al*., 1996) and negatively correlated with oleic acid (Luo *et al*., 2017). For agronomic traits, which have complex genetic architecture and low heritability, their improvement was not remarkable from RS1 to RS3, because the field selection was not as accurate as the indoor phenotype selection performed in RS4 and RS5, which leads to improved agronomic traits from RS3 to RS5 (Figure 5, Table S6). To increase the selection efficiency of the yield‐related traits, we may replace the mass selection, which is vulnerable to the environment, with full‐sib recurrent selection, half‐sib selection, S_*i*_ selection or even a composite scheme according to their genetic characteristics in future research. The RS selection that we performed in this study will provide insights to explore the novel genetic and breeding value of novel germplasm derived from interspecific crosses by promoting recombination through mass intercrossing and providing lessons for crop genetic improvement, especially yield‐related complex traits.

Previous research showed that intersubgenomic hybrids derived from crosses between natural lines and genome‐replaced/resynthesized lines offer a substantial heterosis potential in *B. napus* and *Brassica juncea* (Fu *et al*., 2012; Gupta *et al*., 2015; Wei *et al*., 2016; Zou *et al*., 2010). The RS subpopulation of the gene pool apparently differed from the traditional *B. napus* subpopulation, and they were grouped into two different genetic clusters with a larger genetic distance between the lines of the RS subpopulation and traditional *B. napus* than that within the traditional *B*. *napus* subpopulation. The genetic distance between the RS lines and Chinese lines and the RS lines and European lines reached 0.84 and 0.89 respectively. With the genetic divergence between the gene pool and traditional *B. napus*, this gene pool is an available resource to explore heterosis in rapeseed breeding. However, a substantial amount of time and manpower was spent on the recurrent selection on the gene pool to improve its breeding value, and some traits and crosses of plants remained difficult to test during the selection. We are attempting to construct a genomic prediction model for the new‐type *B. napus* using high‐throughput sequencing data and rigorous field experimental design, and our goal is that it can be applied to complex trait selection to more efficiently improve the breeding value of the gene pool and explore the subgenomic heterosis of the hybrids derived from the new‐type *B. napus*. Therefore, reconstituting the genome of *B. napus* with genomic‐based knowledge and modern technology will promote rapid genome evolution and improvement in mere decades instead of thousands of years.

## Materials and methods

### Developing dominant genic male sterile lines of the new‐type *B. napus*

To form a dynamic gene pool of the new‐type *B. napus* (A^r^A^r^C^c^C^c^), which could incorporate the genetic diversity of two complementary G3 subpopulations, particularly the Poly‐A^r^ and Poly‐C^c^ subpopulations and to conduct multiple rounds of recombination among different G3 individuals for recurrent selection, we introduced a dominant genic male sterile system, which has advantages compared with a recessive male sterile system: (i) the fertility of fertile plants is pure, and (ii) the progenies of male sterile plants always segregate 1:1 for the dominant male sterile allele (Sorrells and Fritz, 1982). The DGMS gene has been successively applied in the recurrent selection of wheat (Deng and Gao, 1980; Xia *et al*., 2017) and *B. napus* (Shen *et al*., 2013; Zhou and Wu, 1995). We used an elite breeding line of *B. napus*, RS1046AB (Msmsrfrf), with a trait of dominant genic male sterility (DGMS) (Li *et al*., 1985; Liu *et al*., 2008) as the tool to facilitate free intercrossing among the new‐type *B. napus* lines rather than manually intercrossing. The RS1046AB line was first crossed with a hexaploid (A^r^A^r^B^c^B^c^C^c^C^c^), and the sterile pentaploid (A^r^A^n^B^c^C^c^C^n^) that resulted was crossed with a pentaploid (A^r^A^r^B^c^C^c^C^c^) created by Xiao *et al*. (2010). The healthy sterile progenies were crossed with three selected G2 lines (Zou *et al*., 2010) with 80% of the A^r^/C^c^ introgression twice, and new‐type *B. napus* lines with DGMS characteristics (Msmsrfrf) were finally developed (Figure S3) with progenies that always segregated sterile and fertile plants in a ratio of 1:1.

### The process of recurrent selection to create a dynamic gene pool of the new‐type *B. napus*

The new‐type *B. napus* DGMS lines with 804 F_3_ lines representing the Poly‐A^r^ subpopulation (Zou *et al*., 2018) and 857 F_4_ lines representing the Poly‐C^c^ subpopulation (Xiao *et al*., 2010) were used to construct a basic population for recurrent selection (RS). Those two subpopulations contained a total exogenous introgression from 74 accessions of *B. carinata* and 122 accessions of *B. rapa*. We adopted the simplest mass selection scheme (Sorrells and Fritz, 1982) to improve the population of the new‐type *B. napus* by constructing a novel breeding gene pool. The DGMS lines were interplanted with selected inbred G3 lines in the field within a net shed with the latter serving as the supplier of pollen, and bees serving as pollinators (Figure 1). During each round of RS, the DGMS lines generated fertile and sterile plants. We removed the fertile plants with unfavourable agronomic traits, such as disease susceptibility, lodging, malformation and small size, during the developmental stage to avoid their pollen dispersal. Favourable fertile and sterile plants were labelled during flowering time, and only the best plants with good field performance, such as lodging resistance, health and high fecundity, were harvested to further phenotype the single plants. For the first three rounds of selection, the agronomic traits were solely selected in the field through visual inspection, but for the last two rounds of selection, we performed agronomic trait selection through a combination of outdoor visual inspection and indoor phenotyping. We selected and grew the sterile plants with relatively good pod traits (pod density, seed number per pod and seed weight) and seed quality (oil content, erucic acid content and glucosinolate content) for the next round of RS. The relatively superior fertile plants were selected to breed inbred and DH lines and were used as the donor for the RS. The seeds from sterile plants were obtained by successful pollination from other fertile plants. Therefore, each plant in the early generations of this recurrent selection (RS) population contained the subgenomes of the Poly‐A^r^ or Poly‐C^c^ subpopulations. After five rounds of RS, most plants of the RS population at advanced generations were expected to have combined subgenomes of the two polymorphic subpopulations and to have realized extensive outcrossing of the two polymorphic subpopulations. Thus, it is likely that abundant genetic recombination and introgression of variation occurred during this process. Eventually, we constructed a highly heterogeneous breeding gene pool of the new‐type *B. napus* with exogenous introgression from 74 accessions of *B. carinata* and 122 accessions of *B. rapa* (Figure 1). Simultaneously, 884 inbred lines and 893 DH lines were bred from the gene pool.

### Plant materials to evaluate phenotypic improvement and genetic variation in the recurrent selection population

To evaluate trait improvement through recurrent selection, a total of 240 lines each consisting of 80 plants randomly selected from the plants derived from the first, third and fifth round of recurrent selection (RS), which were correspondingly recorded as the RS1, RS3 and RS5 subpopulations, respectively, were planted in a field at the Huazhong Agricultural University (Wuhan, China) in 2014. Because each plant from the RS population was a heterozygote with limited seeds, each line was just performed with one plot with no replication. Each plot was 1.8 m^2^, and 36 plants were planted in three rows with a distance of 30 cm between the rows and 17 cm between the individuals. Two plants of each line were randomly selected to test their genotype. Three to five fertile plants of each line were randomly selected at the flowering time to test their agronomic traits and seed quality traits. The agronomic traits tested included pod density (PD), which was counted as the number of pods per cm in the primary inflorescence, seed number per pod (SN), and thousand seed weight (TSW). Seed quality traits were tested using a Foss NIR Systems 5000 with near‐infrared spectroscopy (NIR) as previously described (Gan *et al*., 2003). The genetic improvement of a trait from the i^th^ round to the nth round was ∆*G* = (*μ*_n_–*μ*_i_)/*μ*_i_, where *μ*_i_ and *μ*_n_ are the mean values of the RSi and RSn populations respectively.

### DNA extraction and molecular marker assays

To evaluate the genetic diversity of the novel recurrent selection population, two plants were randomly selected from each of the 80 lines from the RS3 and RS5 subpopulations. Their parental species, 55 accessions of *B. carinata*, 55 accessions of *B. rapa* and 56 accessions of traditional *B. napus* (abbreviated as T‐*B. napus*) were used as controls (Table S1). All the DNA samples were extracted from young leaves using a modified version of the CTAB method as previously described (Stewart and Via, 1993). Eighty pairs of SSR primers covering 19 chromosomes of the A and C genome were selected from the markers mapped in the genetic map of the BnaTN DH population (Luo *et al*., 2017) and the BcYW DH population (Guo *et al*., 2012), and two Indel markers (Larkan *et al*., 2013) were used in this study (Table S2); most of those markers located in the QTL regions accounted for important traits. An ABI 3500xL Genetic Analyzer analysed the PCR products. Fifty‐one new‐type *B. napus* DH lines that have relatively better agronomic performance and bred from different RS lines and 11 traditional *B. napus* lines (including the original parent HS3 of the new‐type *B. napus*) (Table S1) were genotyped by sequencing using the double‐digested restriction‐site associated DNA (ddRAD) sequencing method (Chen *et al*., 2013; Wu *et al*., 2016) but with paired‐end (PE) reads of 150 bp. This resulted in a total of 164 billion paired‐end reads (43 Gb of sequence) with an average depth of 18.64 and coverage of 1.84%.

### Genotype data analysis

The sequences of the 82 SSR primers and Indel primers were BLASTed to the reference genome of *B. napus* ‘Darmor**‐** *bzh*’ (version 4.1) (Chalhoub *et al*., 2014), *B. rapa* v2.5 (Wang *et al*., 2011), *B. oleracea* (Parkin *et al*., 2014) and *B. nigra* ‘YZ12151’ (Yang *et al*., 2016). The physical position of each marker was determined based on the BLAST hits, its position on the genetic map and the allele distributions in different subpopulations (Table S2, Figure S4): (i) when the alleles of a marker were detected only in *B. napus* or *B. carinata* and it was aligned to the C genome of the genetic map and reference genome, the marker was considered to be a C genome‐specific locus (Figure S4a); (ii) When the alleles of a marker were detected only in *B. napus* or *B. rapa*, and it was aligned to the A genome of the genetic map and reference genome, the marker was considered to be an A genome‐specific locus (Figure S4b); (iii) When the alleles of a marker were divided into two loci that could be detected in *B. napus* /*B. carinata* and *B. napus* /*B. rapa* and positioned to the C genome and A genome of the genetic map and reference genome, respectively, the marker amplified a C genome‐specific locus and an A genome‐specific locus (Figure S4c), and (iv) When the allele of a locus was detected in *B. napus*,* B. rapa* and *B. carinata,* and aligned to the A and B genomes of the genetic map and reference genome, the locus was considered as an A/B genome‐specific locus (Figure S4d). We calculated the introgression of the parental alleles in the new‐type *B. napus* according to the specificity of the alleles from different parental species. For the A genomic loci, if an allele specially appeared in *B. rapa*, it was recorded as a *B. rapa* (A^r^) specific allele, and if an allele specifically appeared in traditional *B. napus*, it was recorded as a traditional *B. napus* (A^n^) specific allele (Figure S4b,c,d). For the C genomic loci, if an allele only appeared in *B. carinata*, it was recorded as a *B. carinata* (C^c^) specific allele, and if an allele only appeared in traditional *B. napus*, it was recorded as a traditional *B. napus* (C^n^) specific allele (Figure S4a). Alleles that did not appear in the parental species, but specifically appeared in the new‐type *B. napus* lines, were recorded as novel alleles in the new‐type *B. napus*. When calculating the genetic diversity of the new‐type *B. napus* and traditional *B. napus* in separate A and C genomes, the A/B genome‐specific loci were designated as A genome markers. Statistics, including the number of alleles per locus, genetic diversity and heterozygosity, were determined in terms of three data sets, specifically, the total loci, A genomic loci and C genomic loci using PowerMarker version 3.25 (Liu and Muse, 2005). Nei's genetic distance (Nei, 1972) was calculated and used for the unrooted phylogeny reconstruction in the Neighbour‐Joining method using PowerMarker with the obtained tree visualized via MEGA 6.0 (Tamura *et al*., 2007).

Genotype calling of the 51 new‐type *B. napus* DH lines and 11 traditional *B. napus* (including HS3, the original *B. napus* parent of the new‐type *B. napus*) was performed as described by Wu *et al*. (2016) with modifications. Clean reads were mapped to the reference genome with Bowtie2 v2.3.4 (https://sourceforge.net/projects/bowtie-bio/files/bowtie2/2.3.4/) using the default parameters. SAMtools v1.8 (https://sourceforge.net/projects/samtools/files/samtools/1.8/) was used to convert and sort mapping results into the BAM format. Duplicate reads were filtered using the Picard v2.12.1 package (https://github.com/broadinstitute/picard/tree/2.12.1), and the indexing for the BAM file was constructed using SAMtools v1.8. SNP detection was performed using GATK v4.0.3.0 (https://github.com/broadinstitute/gatk/releases/tag/4.0.3.0), and the details were as follows: (i) Using the HaplotypeCaller package runs per‐sample to generate an intermediate GVCF file; (ii) Using the CombineGVCFs package to combine per‐sample GVCF files into a multi‐sample GVCF file; (iii) Using the GenotypeGVCFs package to perform joint genotyping on more samples pre‐called using HaplotypeCaller and (iv) Using the VariantFiltration package to filter variant calls based on INFO and/or FORMAT annotations. The reference genomes of *B. napus* ‘Darmor‐*bzh*’ (version 4.1) (Chalhoub *et al*., 2014), *B. rapa* v2.5 (Wang *et al*., 2011), *B. oleracea* v2.1 (Parkin *et al*., 2014) and *B. nigra* ‘YZ12151’ (Yang *et al*., 2016) were used for the genotype calling to detect more species‐specific markers. First, a set of A and C genomic SNP/Indel markers were obtained using the *B. napus* genome ‘Darmor‐*bzh*’ as a reference. Second, a set of A, B and C genomic SNP/Indel markers were obtained using the ‘*B. rapa* v2.5, *B. oleracea*, and *B. nigra*’ genome as a reference. Third, the markers obtained based on the ‘*B. rapa* v2.5, *B. oleracea*, and *B. nigra*’ genome were BLASTed to the markers obtained based on the *B. napus* genome using TBtools software (https://github.com/CJ-Chen/TBtools/releases) with the unmapped markers recorded as additional detected *B. rapa‐* and *B. carinata*‐specific markers. Fourth, for the B genomic marker, when all 11 traditional genotypes of the *B. napus* accessions were missing, the marker was recorded as a *B. carinata*‐specific B genomic marker. Markers with missing rates less than 50% and HS3 as the reference were used to assess the specific introgressions of the DH lines from *B. rapa* and *B. carinata*, and when the genotype of a DH line was different from that of HS3, alleles of the DH lines of the marker that were different from those of HS3 were recorded as allelic variation that was introgressed from *B. rapa* and *B. carinata*.

## Author contributions

DDH performed the research, analysed the data and wrote the manuscript; LLC and YYC performed the recurrent selection of the gene pool; WSZ and SHC contributed to phenotyping; YKZ analysed the GBS data; YDS helped with the molecular marker assays; JXS helped with the selection of the gene pool; JLM contributed plant materials and provided ideas and suggestions for the project design and manuscript revision; JZ designed the research, analysed the data and revised the manuscript. All authors read and approved the final manuscript.

## Conflict of interest

The authors declare that they have no competing interests.

## Supporting information


**Figure S1** Genotype of the new‐type *Brassica napus* gene pool and traditional *B. napus*.

**Figure S2** Distribution of the specific introgressions of new‐type *Brassica napus* DH lines. (a), (b), (c), (d) and (e) show the distribution of the specific introgression of new‐type *B. napus* DH lines with markers detected using the *B. napus* A genome, *B. rapa* genome, *B. napus* C genome, *Brassica oleracea* genome and *B. nigra* genome as a reference, respectively. The DH lines were arranged according to the portion of specific introgression. Gray lines indicate that the allele detected in the new‐type *B. napus* DH lines was the same as that in HS3, and the red lines indicate that the allele detected in the new‐type *B. napus* DH lines was different from that in HS3.

**Figure S3** Introducing the trait of dominant genic male sterility (DGMS) from traditional *Brassica napus* to the new‐type *B. napus*.

**Figure S4** Demonstration of the detection of the alleles from different parental species using SSR markers. (a) A pair of SSR primers amplified a C genome‐specific locus. (b) A pair of SSR primers amplified an A genome‐specific locus. (c) A pair of SSR primers amplified an A genome‐specific locus and a C genome‐specific locus. (d) A pair of SSR primers amplified an A genome‐specific locus and a B genome‐specific locus.

**Table S1** Name and geographical origin of the accessions used for genotyping.

**Table S2** SSR primers and Indel primers used to genotype the 486 accessions.

**Table S3** B genomic signals identified in the new‐type *B. napus* DH lines revealed by SNP/Indel markers through genotyping by sequencing.

**Table S4** Annotation genes located in the 160 kb B genomic region.

**Table S5** Seed quality and agronomic traits of the sterile plants harvested in each round of recurrent selection.

**Table S6** Seed quality and agronomic traits of the S1, S3 and S5 subpopulations investigated in the same environment in 2014.
