# Supplementary figures and images for: Reconstituting the genome of a young allopolyploid crop, Brassica napus, with its related species
Source: Plant Biotechnol J. 2019 Jan 7;17(6):1106–18. doi: 10.1111/pbi.13041 (PMC6523605; doi:10.1111/pbi.13041)

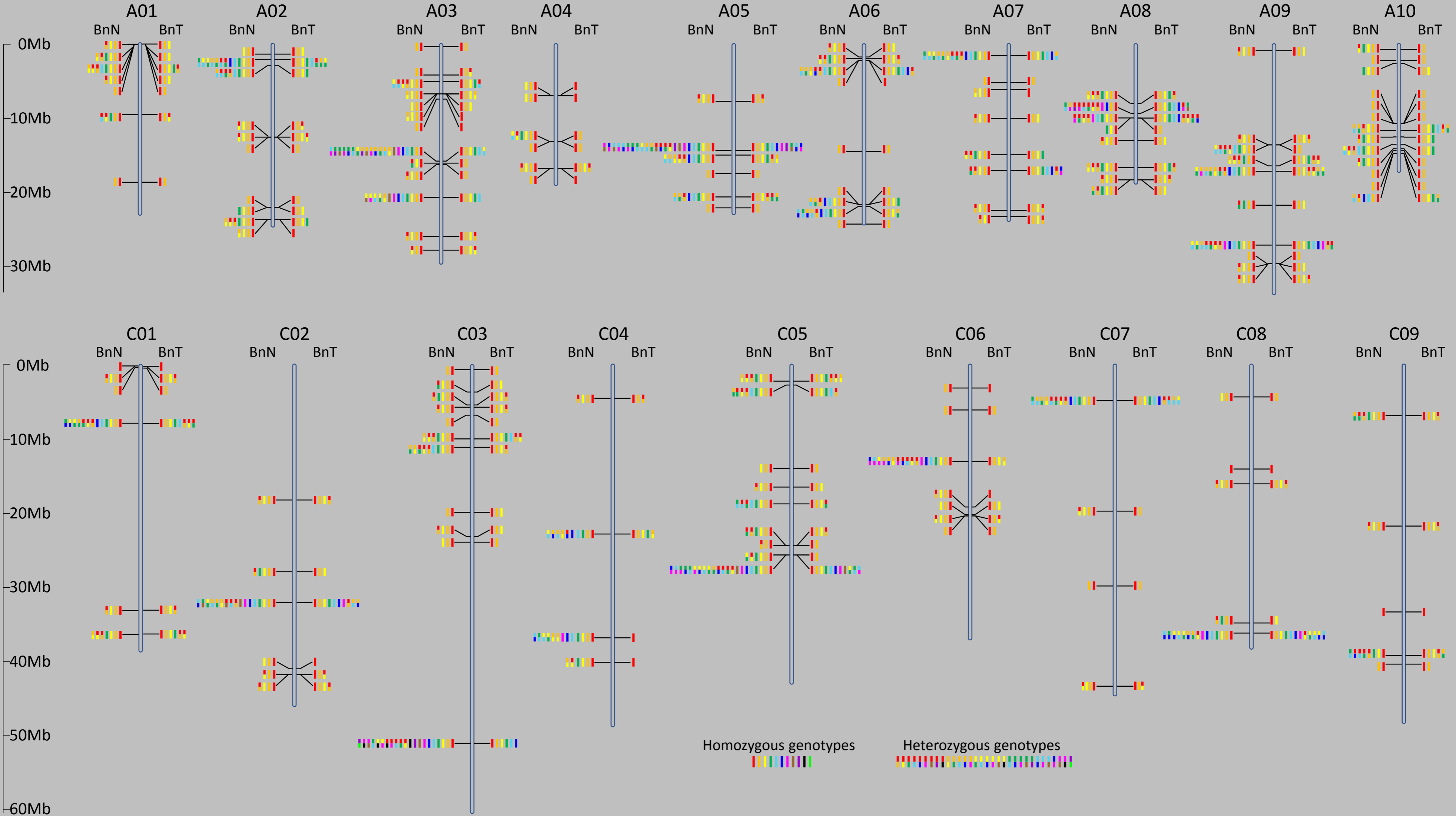

Supplement: Supplementary file 1 — Figure S1 Genotype of the new‐type Brassica napus gene pool and traditional B. napus. [file PBI-17-1106-s005.pdf]

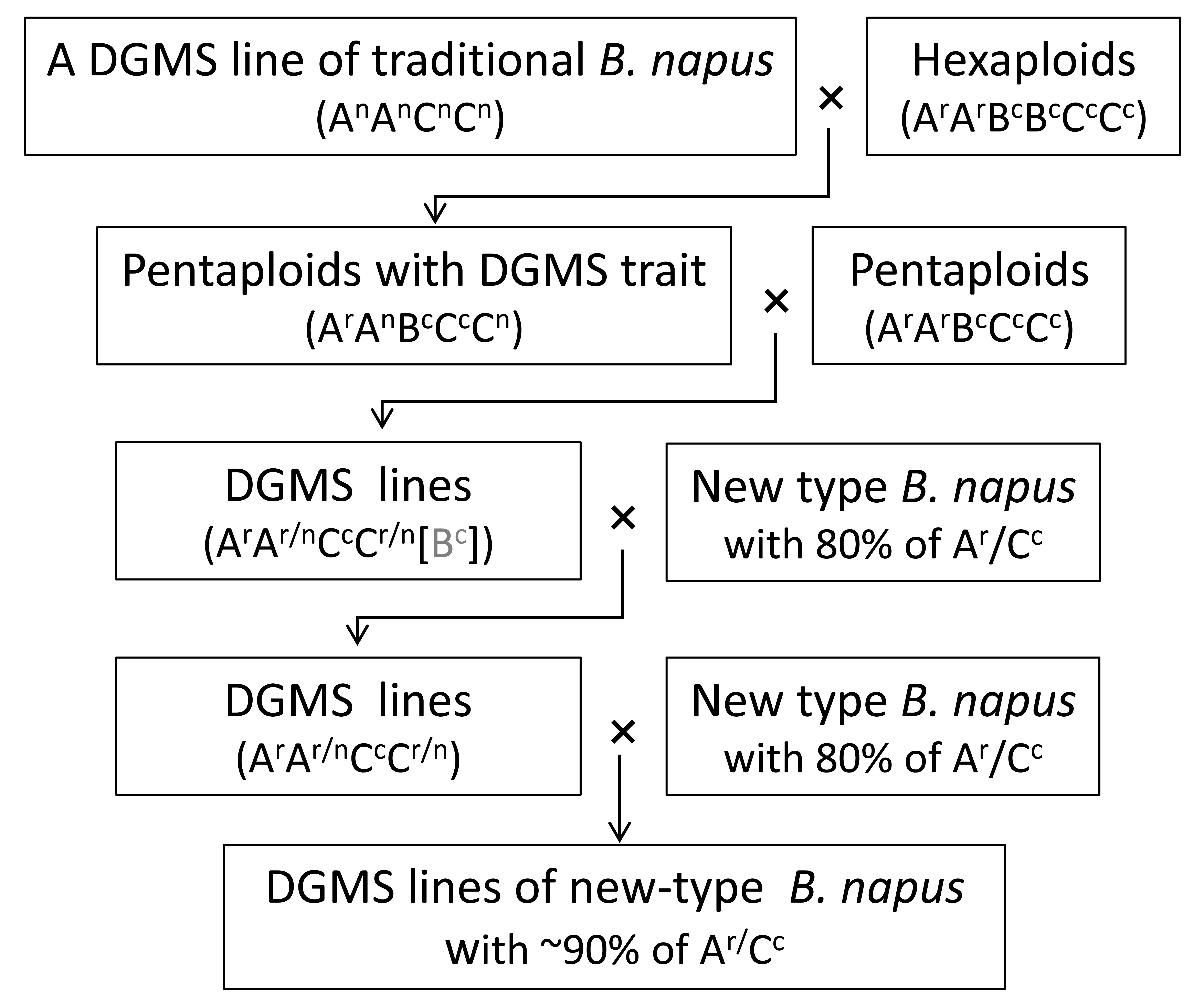

Supplement: Supplementary file 3 — Figure S3 Introducing the trait of dominant genic male sterility (DGMS) from traditional Brassica napus to the new‐type B. napus. [file PBI-17-1106-s007.jpg]

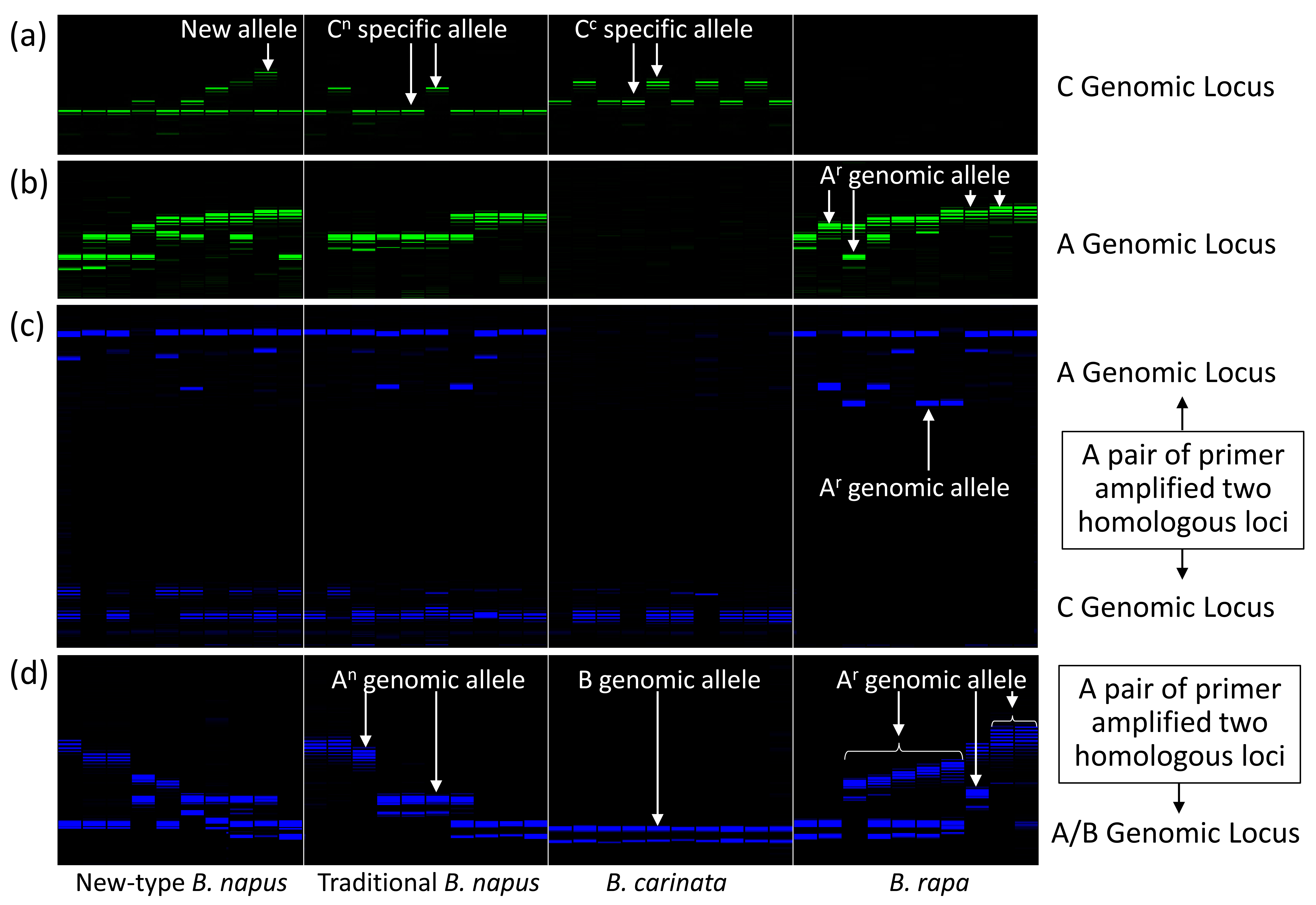

Supplement: Supplementary file 4 — Figure S4 Demonstration of the detection of the alleles from different parental species using SSR markers. (a) A pair of SSR primers amplified a C genome‐specific locus. (b) A pair of SSR primers amplified an A genome‐specific locus. (c) A pair of SSR primers amplified an A genome‐specific locus and a C genome‐specific locus. (d) A pair of SSR primers amplified an A genome‐specific locus and a B genome‐specific locus. [file PBI-17-1106-s009.jpg]
