# Supplementary material for: Reconstituting the genome of a young allopolyploid crop, Brassica napus, with its related species
Source: Plant Biotechnol J. 2019 Jan 7;17(6):1106–18. doi: 10.1111/pbi.13041 (PMC6523605; doi:10.1111/pbi.13041)
Supplement: Supplementary file 2 — Figure S2 Distribution of the specific introgressions of new‐type Brassica napus DH lines. (a), (b), (c), (d) and (e) show the distribution of the specific introgression of new‐type B. napus DH lines with markers detected using the B. napus A genome, B. rapa genome, B. napus C genome, Brassica oleracea genome and B. nigra genome as a reference, respectively. The DH lines were arranged according to the portion of specific introgression. Gray lines indicate that the allele detected in the new‐type B. napus DH lines was the same as that in HS3, and the red lines indicate that the allele detected in the new‐type B. napus DH lines was different from that in HS3. [file PBI-17-1106-s008.pdf]

(a)

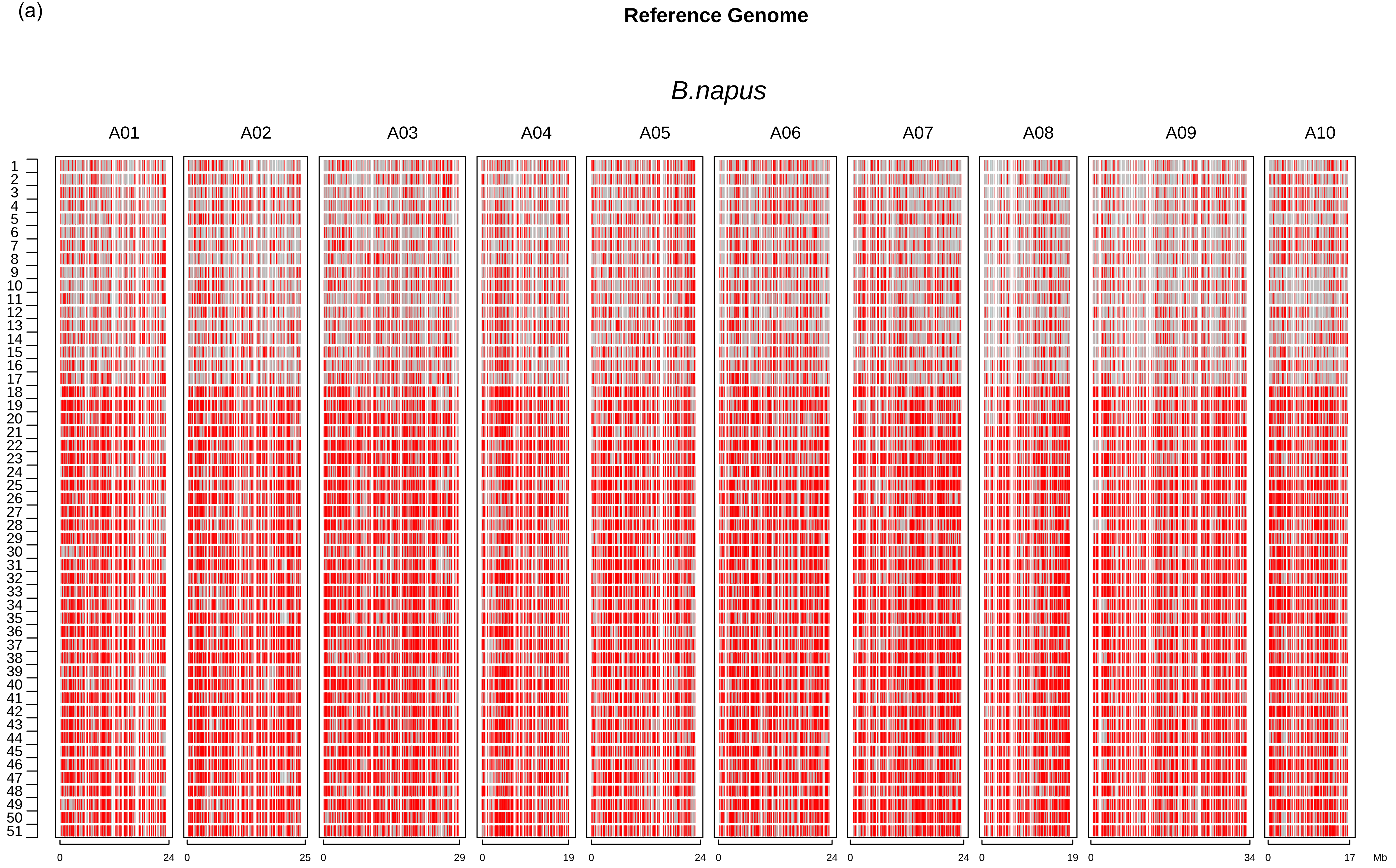

DH lines were arranged according to the portion of specific introgression.
Gray lines indicate that the allele detected in the new-type B. napus DH lines was the same as that in HS3.
Red lines indicate that the allele detected in the new-type B. napus DH lines was different from that in HS3.

(b)

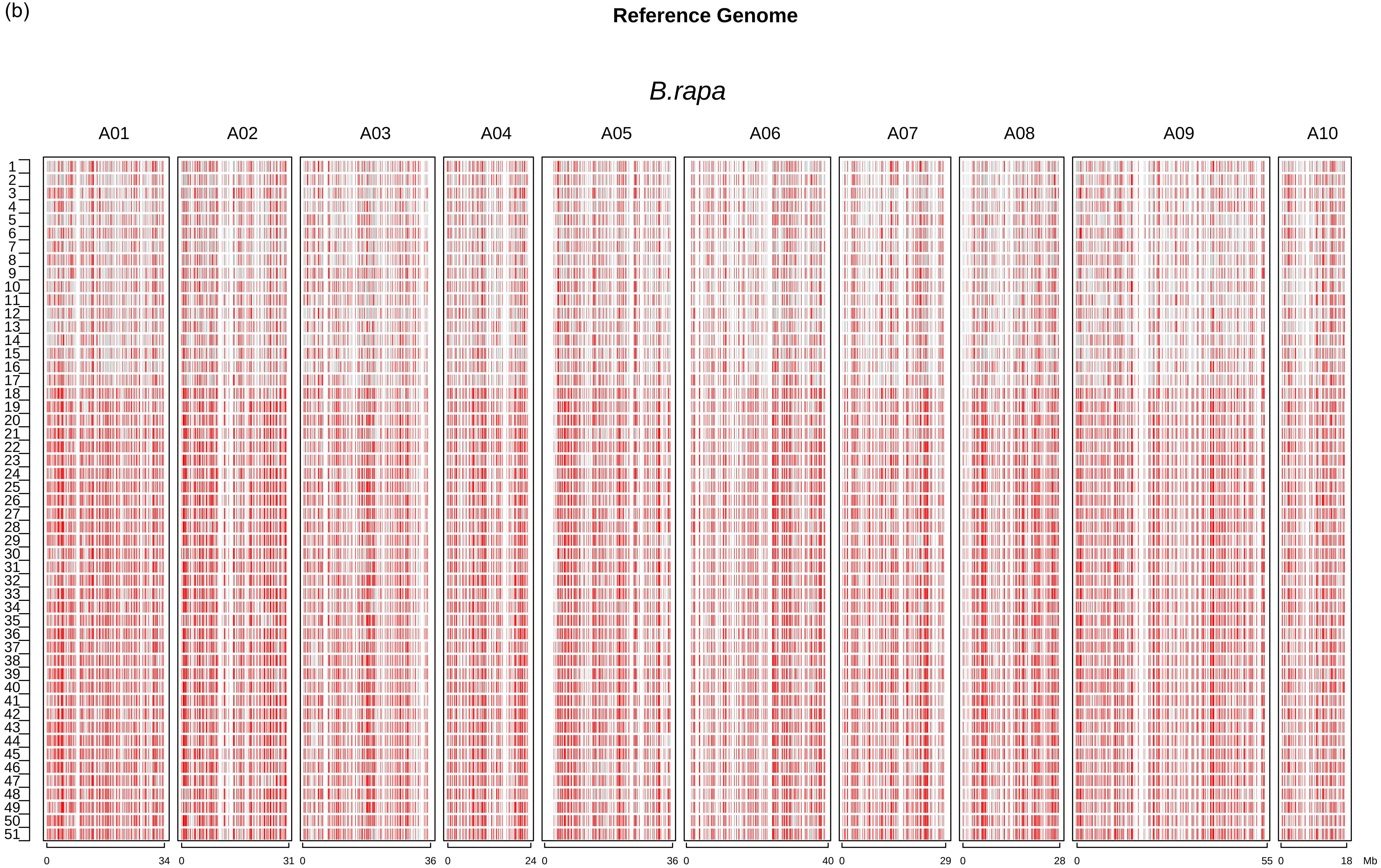

DH lines were arranged according to the portion of specific introgression.
Gray lines indicate that the allele detected in the new-type B. napus DH lines was the same as that in HS3.
Red lines indicate that the allele detected in the new-type B. napus DH lines was different from that in HS3.

(c)

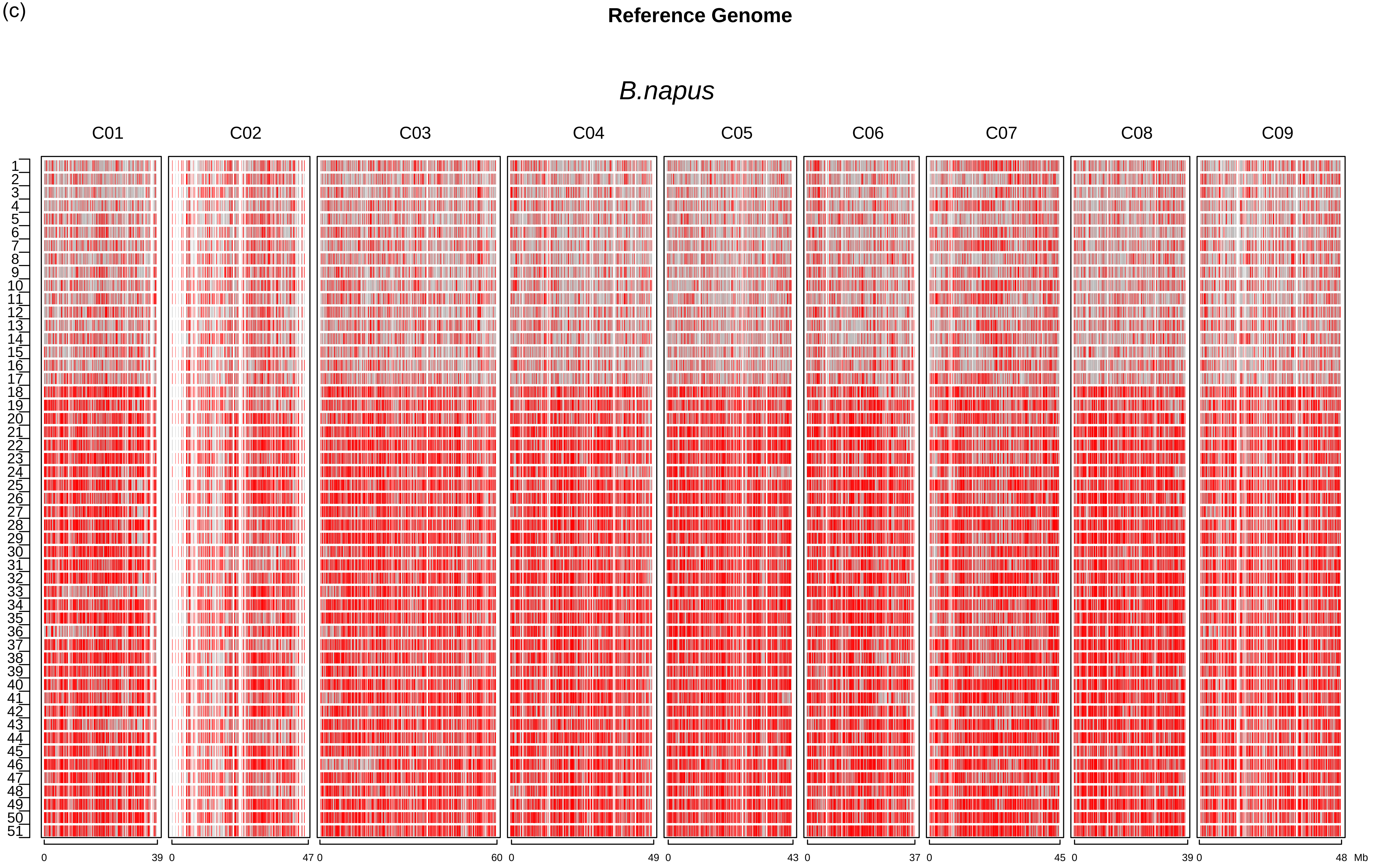

DH lines were arranged according to the portion of specific introgression.
Gray lines indicate that the allele detected in the new-type B. napus DH lines was the same as that in HS3.
Red lines indicate that the allele detected in the new-type B. napus DH lines was different from that in HS3.

(d)

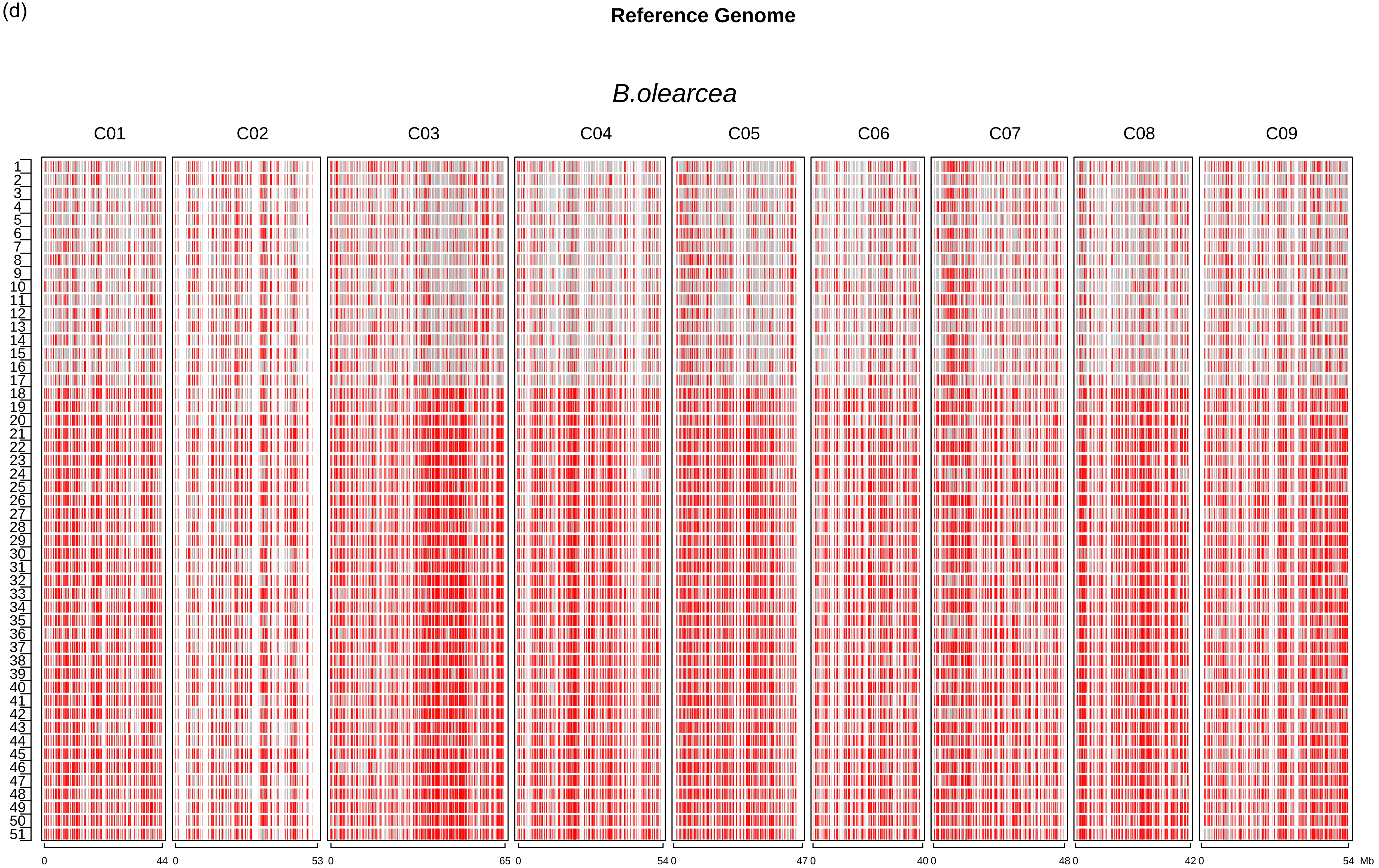

DH lines were arranged according to the portion of specific introgression.
Gray lines indicate that the allele detected in the new-type B. napus DH lines was the same as that in HS3.
Red lines indicate that the allele detected in the new-type B. napus DH lines was different from that in HS3.

(e)

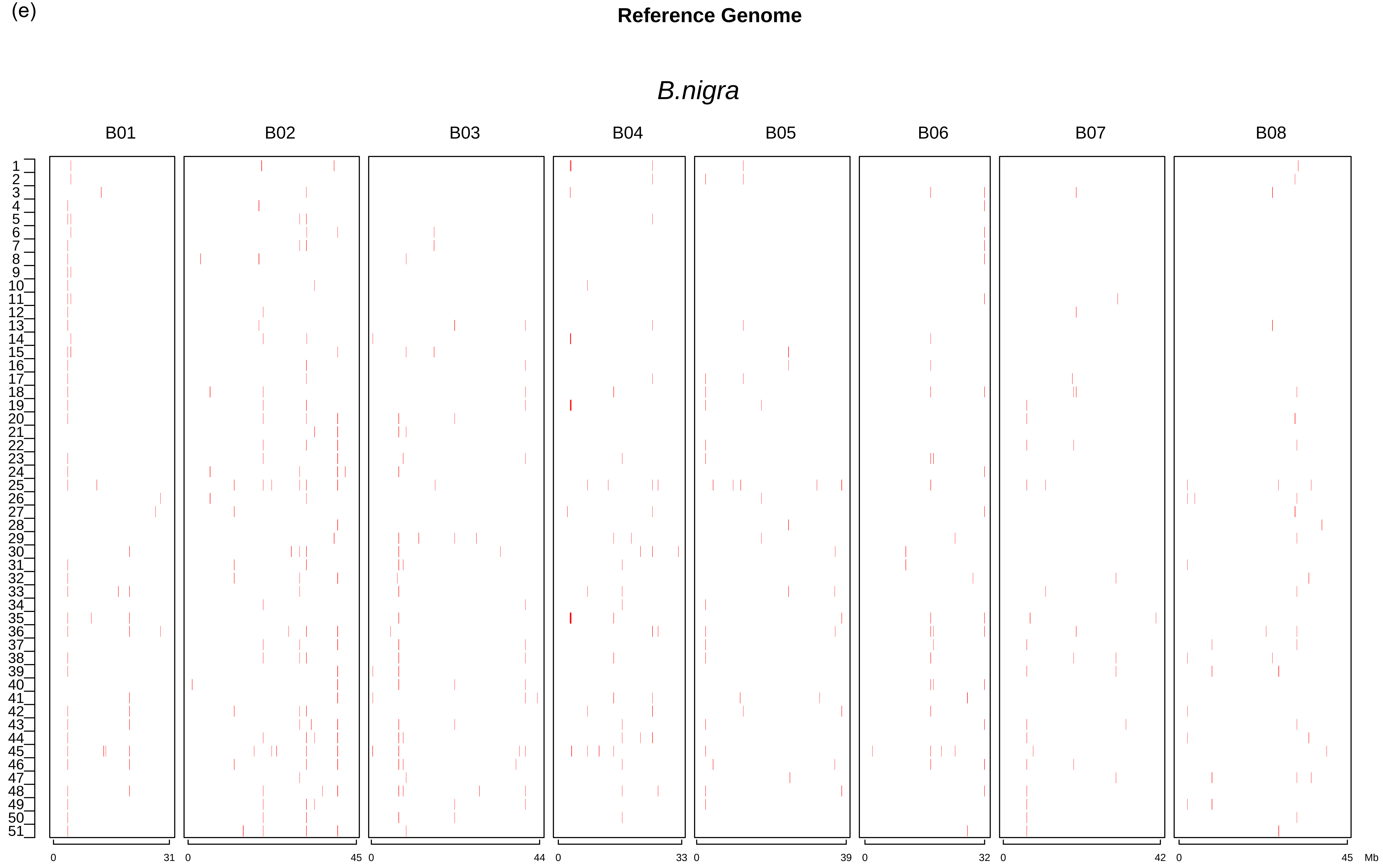

DH lines were arranged according to the portion of specific introgression.
Red lines indicate that the allele detected in the new-type B. napus DH lines was different from that in HS3.
