# Supplementary material for: Reconstituting the genome of a young allopolyploid crop, Brassica napus, with its related species
Source: Plant Biotechnol J. 2019 Jan 7;17(6):1106–18. doi: 10.1111/pbi.13041 (PMC6523605; doi:10.1111/pbi.13041)
Supplement: Supplementary file 10 — Table S6 Seed quality and agronomic traits of the S1, S3 and S5 subpopulations investigated in the same environment in 2014. [file PBI-17-1106-s006.docx]

**Table S6 Seed quality s and agronomic traits of the S1, S3 and S5 subpopulations investigated in the same environment in 2014.**

| Traits | S1 | |  | S3 | |  | S5 | | | |
| --- | --- | --- | --- | --- | --- | --- | --- | --- | --- | --- |
|  | **Mean** | **Max** |  | **Mean** | **Max** |  | **Mean** | **Max** | |  |
| Oil content | 44.19 ± 3.47* | 51.5 |  | 44.44 ± 3.54 | 51.4 |  | 44.91 ± 2.92 | | 52.0 | |
| Eurcic acid content (%) | 9.36 ± 7.88 | 31.6 |  | 3.86 ± 6.49 | 30.5 |  | 0.99 ± 1.99 | | 15.8 | |
| Oleic acid (18:1) content (%) | 50.24 ± 9.47 | 67.08 |  | 55.56 ± 8.51 | 68.94 |  | 59.81 ± 3.96 | | 68.36 | |
| Linoleic acid (18:2) content (%) | 19.06 ± 2.09 | 27.63 |  | 20.10 ± 1.77 | 26.63 |  | 20.63 ± 1.71 | | 28.39 | |
| Glucosinolate content (μmol/g) | 60.90 ± 22.98 | 130.2 |  | 46.21 ± 17.66 | 113.3 |  | 36.19 ± 8.46 | | 82.46 | |
| Thousand seed weight (g) | 3.90 ± 0.65 | 5.67 |  | 3.91 ± 0.68 | 6.36 |  | 4.00 ± 0.58 | | 6.12 | |
| Pod density | 1.05 ± 0.21 | 2.11 |  | 1.09 ± 0.22 | 2.53 |  | 1.11 ± 0.19 | | 2.20 | |
| seed number per pod | 21.1 ± 3.90 | 31.4 |  | 20.7 ± 3.95 | 30.8 |  | 21.8 ± 3.60 | | 29.9 | |

*****: standard deviation
